# Conflicts between priming and episodic retrieval: a question of fluency?

**DOI:** 10.1007/s00426-023-01919-4

**Published:** 2024-02-28

**Authors:** Peter Weller, Guillermo Recio, Laura Kaltwasser, Hadiseh Nowparast Rostami, Birgit Stürmer, Werner Sommer

**Affiliations:** 1 Humboldt-Universität zu Berlin, Berlin, Germany; 2 Universitat de Barcelona, Barcelona, Spain; 3 Berlin School of Mind and Brain, Humboldt-Universität zu Berlin, Berlin, Germany; 4 International Psychoanalytic University, Berlin, Germany

## Abstract

Human memory consists of different underlying processes whose interaction can result in counterintuitive findings. One phenomenon that relies on various types of mnemonic processes is the repetition priming effect for unfamiliar target faces in familiarity decisions, which is highly variable and may even reverse. Here, we tested the hypothesis that this reversed priming effect may be due to a conflict between target fluency signals and episodic retrieval processes. After replicating the reverse priming effect, three different manipulations were effective in diminishing it. We suggest that each of these manipulations diminished the ambiguity regarding the source of priming-induced fluency of target processing. Our findings argue against a strictly independent view of different types of memory.

**Supplementary Information:**

The online version contains supplementary material available at 10.1007/s00426-023-01919-4.

## Introduction

Human memory consists of various distinct processes. Two phenomena that are frequently investigated in human memory are repetition priming, which can be seen in faster responses to stimuli that have been previously encountered (Roediger & McDermott, 1993), and recognition memory where participants consciously retrieve the study context of previously presented stimuli (Yonelinas, 2002). Numerous lines of evidence have led to the position that memory effects arising from repetition priming and those arising from recognition memory reflect distinct processes. For example, repetition priming is spared in amnesic patients despite deficits in recognition performance (Graf et al., 1984; Warrington & Weiskrantz, 1974). This dissociation is further supported by neural evidence showing that repetition priming is subserved by cortical areas involved in perceptual and semantic processing, whilst recognition is dependent on the temporal lobe (Squire, 1994). Furthermore, behavioural studies show that factors such as study duration (Voss & Gonsalves, 2010), increased semantic processing, and modality changes between prime and target can have opposite effects on repetition priming and recognition (Richardson-Klavehn & Bjork, 1988).

In contrast, other strands of evidence suggest that repetition priming and recognition memory may not be entirely independent. Modeling simulations of repetition priming and recognition decisions have led to a of these processes (Berry et al., 2008, 2012), and double dissociations between repetition priming and recognition memory in amnesic patients have been challenged (Ostergaard & Jernigan, 1993). In a seminal study, Jacoby and Whitehouse (1989) demonstrated that repetition priming and recognition memory involve similar signals when both are combined in a single procedure. In their study, words from an initial learning period were presented to participants as targets for recognition decisions. These recognition targets were either primed by the same target word or by a different word. In addition, the prime presentation duration was manipulated causing prime words in one condition to be processed outside of awareness. The authors predicted that priming the target word would facilitate its processing, resulting in participants experiencing it as more familiar—irrespective of its actual familiarity status—and therefore more likely to be judged as old. Their results supported the hypothesis that false alarms increased for primed new target words. This effect was limited to trials where participants were unaware of the prime. The authors reasoned that priming target words increases their fluency—defined as the speed or ease with which information can be extracted from the target—inducing a subjective sense of familiarity for new words (Jacoby & Dallas, 1981). Furthermore, when participants were aware of the prime, the increased processing fluency for new targets was more easily attributable to its previous presentation as a prime. However, when the prime was presented outside of awareness, the experience of processing fluency was misattributed to the initial learning phase of the experiment causing participants to judge new targets as old.

The present study explores the influences of repetition priming and recognition memory on processing fluency in the context of unfamiliar faces, which demonstrate highly variable priming effects. For faces of celebrities or newly learned faces, there are very consistent priming effects, between 100 and 200 ms (e.g. Dörr et al., 2011; Engst et al., 2006; Herzmann & Sommer, 2010; Herzmann et al., 2010; Pfütze et al., 2002; Schweinberger et al., 1995). These effects are not confined to faces but are also seen for other materials, such as names (Pfütze et al., 2002), words, or pictures of objects (for a review see Roediger & MacDermott, 1993). In contrast, priming effects for unfamiliar faces are generally smaller (Herzmann & Sommer, 2010; Herzmann et al., 2004, 2010; Schweinberger et al., 1995), and may fail significance (Herzmann & Sommer, 2007; Pfütze et al., 2002) or may even become negative (Kaltwasser et al., 2014).

In the present study, we take the findings of Kaltwasser et al. (2014) as a starting point to further explore how different memory processes may interact. Specifically, we investigate the relation between prime-induced target processing fluency and episodic retrieval processes, and the conditions that facilitate conflict between these two types of memory.

In the study by Kaltwasser et al. (2014), participants first memorised a set of 10 previously unfamiliar faces, which were subsequently used as familiar targets in a familiarity decision task, requiring an “old” response. These learned faces were randomly mixed with new, unfamiliar target faces, requiring a “new” response. Preceding each familiar or unfamiliar target face a prime face was presented, which was either the same face as the target (primed) or a different face (unprimed). Furthermore, an additional face was inserted between the prime and target faces serving as a mask for the prime. All masking faces were unfamiliar, never shown twice, and not related to prime or target faces. The behavioural results showed a standard priming effect for familiar target faces, with participants responding faster to primed targets than to unprimed targets. Unexpectedly however, responses to unfamiliar target faces were slower when primed by the same face than when preceded by a different face. We hereafter refer to this finding from Kaltwasser et al. (2014) as the reversed priming effect.

We hypothesise that this reversed priming effect for unfamiliar target faces is a result of a conflict between memory signals. We propose that there are at least two processes involved in generating these signals, the first process arises from an increase in processing fluency for repeated targets, and the second is an episodic retrieval process, which involves the conscious episodic retrieval of the previous study context and the learned faces associated to that context. Specifically, repetition priming of unfamiliar target faces would have facilitated a “familiar” response because participants misattribute the increased fluency that arises during target processing to the study episode, whilst the results from episodic retrieval processes may have facilitated an “unfamiliar” response as the target is not part of the previously learned memory set. These opposing tendencies would have induced a conflict which may explain the slowing of correct reaction times to primed unfamiliar targets. Conflicts between different aspects of memory have been observed in the domains of episodic memory retrieval (Anderson et al., 1994), across semantic representations (Rose & Abdel Rahman, 2017) and amongst items held in working memory (Jonides & Nee, 2006), however less is known about how conflict effects may emerge between different types of memory. In the present study, we aimed to replicate and elucidate the reversed priming effect by testing this mnemonic conflict hypothesis. We manipulated several aspects of the experimental design that may have contributed to the effect, namely, the contribution of the face mask stimulus presented between the prime and the target and the role of episodic retrieval processes.

## The role of mask stimuli intervening between prime and target

Kaltwasser et al. (2014) design differed from most previous studies in that it employed a backward-masking procedure, whereby during the interval between the prime and target faces, an unfamiliar face, the “mask”, was briefly but recognisably presented. The backward face masking procedure was also used by Dörr et al. (2011), who found that presenting an unfamiliar face mask between prime and target in a semantic decision task attenuated the priming effect in reaction times, indicating that the backward masking procedure disrupts prime processing to some degree. We propose that this disruption in prime processing may contribute to the reversed priming effect.

But how might a disruption of prime processing lead to a significant slowing in reaction times for primed trials? We suggest that, as in the subliminal condition of Jacoby and Whitehouse (1989), the presentation of the face mask momentarily caused some ambiguity in regard to the source of the fluency signal generated by the target face. An intervening face mask may have a disruptive effect on the encoding and/or retrieval of source information associated with the prime stimulus, which is required to correctly attribute processing fluency to the prime and not to the encoding phase. Many theories view priming as operating via the creation and retrieval of episodic memory traces (Bodner & Masson, 1997; Brunel et al., 2009; Hommel, 1998). From such a perspective, presentation of the prime stimulus establishes an episodic memory trace, which is then retrieved upon the presentation of the target stimulus, resulting in speeded reaction times to the stimulus whenever the memory trace and the target representation match. A disruption of source information associated with the prime by an intervening mask would result in some ambiguity regarding the origin of the increased feeling of familiarity for primed unfamiliar targets, whereas for unmasked primes, the source of the feeling of familiarity is readily available. Thus, one aim of the present experiments was to investigate the role of the intervening face mask in causing the reversed priming effect.

## The role of episodic retrieval

Though the face mask may contribute to the reversal of the priming effect for unfamiliar faces, it is unlikely that this is the only factor. Dörr et al. (2011) found substantial positive priming effects for reaction times to unfamiliar faces of around 70 ms, despite their backward masking procedure. Another difference between their study and the one by Kaltwasser et al. (2014) was the type of familiar face targets. Dörr et al. (2011) required familiarity decision on celebrities versus unfamiliar target faces (Exp. 1), and semantic judgments on a single set of famous faces (Exp. 2). Usually, participants are exposed to famous faces on numerous occasions and in various contexts over many years and thus their corresponding memory representations should be highly consolidated and rich in semantic content. Consequentially, familiarity judgments on famous faces may be carried out not only based on face-related representations or episodic memory, but also by retrieving person-specific semantic information (Burton et al., 1990). In contrast, Kaltwasser et al. (2014) used a set of initially unfamiliar faces, which were studied immediately before the familiarity decision task in order to be used as familiar targets. As these familiar targets lacked semantic detail and were tied to a specific encoding episode, it is likely that in order to carry out the familiarity decision task participants engaged in episodic retrieval, evaluating the target faces in terms of the context in which they had been learned. We propose that the experience of increased processing fluency induced by the prime stimulus interferes with recognition judgements more than with semantic judgements for new faces. As target faces for the recognition judgements came from a newly learned set of faces that lack semantic information, participants are likely to use their memory of perceptual features associated with the learned faces to carry out these judgements. This would result in the prime-induced processing fluency for unlearned faces to be mistaken for familiarity from the encoding episode, causing participants to engage in episodic retrieval. On the other hand, when carrying out semantic judgements, participants search for semantic knowledge of famous faces which they can use to more quickly discount the prime-induced processing fluency, given that semantic memory for famous faces is highly consolidated.

To elucidate these questions, in Experiments 1 and 2, we assessed the role of the intervening face mask on the reversed priming effect by comparing the intervening face mask with a face-sized grey ellipse (Exp. 1) and contextually segregating the mask from the prime and target stimuli (Exp. 2). In Experiment 2, we further assessed the effect of the type of information required to carry out the familiarity decision task by replacing learned faces in the familiar condition with famous faces. In line with the discussion above, we expected these manipulations to attenuate or eliminate the reversed priming effect.

In both experiments, EEG activity was recorded throughout with the aim to assess neurocognitive processes represented by conflict-related event-related potentials (ERPs). However, although the ERPs showed conflict-related components, these were not noticeably affected by our experimental manipulations; we therefore decided to focus on the behavioural effects and report the ERP measurements only in the supplementary material.

## Experiment 1

The aim of Experiment 1 was to determine the role of the intervening face mask in producing the reversed priming effect for unfamiliar target faces. Therefore, we compared a replication of the conditions used by Kaltwasser et al. (2014) where an intervening face mask was present between the prime and target stimulus, with a condition where the intervening mask was replaced with a grey ellipse. In line with the discussion above, we expected the removal of the unfamiliar-face mask to attenuate or eliminate the reversed priming effect.

## Methods

### Participants

The sample consisted of 28 students (50% females, *M* = 27.9 years, range: 22–49, *SD* = 8) who participated either for 8 € per hour or credit points; 95% of the participants were right-handed as assessed by a handedness questionnaire (Oldfield, 1971). All participants reported normal or corrected-to-normal visual acuity, and absence of neurological or psychiatric disorders. Informed consent was obtained from all participants. The study was conducted in accordance with the declaration of Helsinki and had been approved by the ethics committee of the Department of Psychology of the Humboldt University.

### Design

The type of mask between prime and target presentation was manipulated block-wise. Block A aimed to replicate the reverse priming effect by using the same 10 familiar faces, 80 unfamiliar faces, and a novel unfamiliar face mask (160 in total) for each trial (face mask condition). The reason we used ten familiar faces which were repeated often was to replicate the conditions in Kaltwasser et al. (2014). In Block B we presented the same 10 familiar faces and a new set of 80 unfamiliar faces but used a grey ellipse (grey mask condition) between prime and target in each trial instead of a novel unfamiliar face. The order of block presentation was counterbalanced across participants.

### Materials

We used the same stimuli as Kaltwasser et al. (2014). Stimuli consisted of black and white face portraits (50% female) taken from two databases (Endl et al., 1998; Lundqvist et al., 1998), with similar luminance and frontal direction of gaze. All faces showed neutral expressions and were fitted into an elliptical grey frame of 259 by 388 pixels (7.0 by 10.2 cm; 4.0° by 5.8° of visual angle). All faces were devoid of facial hair and glasses. Ten faces which had been rated in a previous pilot study as being the most distinctive of a set of 145 portraits (see Kaltwasser et al., 2014, for additional details), were selected for the familiar conditions. A further 160 faces were selected as primes and targets for the unfamiliar conditions, and 320 faces were used as masks.

### Procedure

The experiment began with a learning phase where the ten faces from the familiar condition were sequentially presented for three seconds each, followed by 12 s of blank screen, during which participants were to write a brief description of the face on a sheet of paper. Following this initial exposure to the set of ten faces, participants then engaged in four cycles of recognition testing where each of the ten learned faces was sequentially presented as old target intermixed with two novel faces, which served as new targets. Participants were to indicate whether or not the face on the screen was a previously studied face with a two-choice button press. Each pair of novel targets was replaced in each round of recognition testing.

Following the learning phase the familiarity decision task started. After reading general instructions, participants first performed five practice trials, and were then presented with specific instructions for the following block. In the block with the face mask, a trial began with the presentation of a fixation cross for 200 ms, followed by the prime stimulus, which remained on the screen for 500 ms and was immediately followed by the mask stimulus, presented for 500 ms. After the mask stimulus there was a blank screen for 800 ms after which the target was shown for 1.5 s. The interval between successive trials was five seconds. No jitter was applied to replicate conditions in Kaltwasser et al. (2014). For half of the trials the target face was one of the ten learned familiar faces, and for the other half, the target face was an unfamiliar face. Unfamiliar targets were presented only once. On half of the trials a target face was either primed, in which case it was a repetition of the prime stimulus, or unprimed, in which case, for familiar targets, the prime was an unfamiliar face, and for unfamiliar targets, the prime was a face from the set of ten familiar stimuli. This resulted in a total of four conditions: Familiar-Primed (FP), Familiar-Unprimed (FU), Unfamiliar-Primed (UP), and Unfamiliar-Unprimed (UU). For grey-masked trials, the same trial procedure was used with the exception that a grey elliptic area served as intervening mask. Each block consisted of 160 trials with 40 trials per condition. Famous faces were repeated eight times, and a new set of 80 unfamiliar faces was used for each block. An additional set of 160 unfamiliar faces were used as masking stimuli in block A. Participants were instructed to attend to the centre of the screen throughout the session and to classify the target faces as either familiar, i.e. belonging to one of the previously learned ten faces, or unfamiliar, ignoring the prime and the mask in their decision. Target face classification was performed by right or left button presses to familiar or unfamiliar stimuli, respectively. Both speed and accuracy were emphasised. Trials within each block were presented in random order and participants were given short breaks every 40 trials. After each block, the instructions for the next block were given.

### Data analysis

All data were trimmed by excluding reaction times < 100 and > 1500 ms. Reaction times < 100 ms cannot be based on stimulus processing, and reaction times > 1.5 s are probably slow guesses or do not reflect optimal behaviour, where people do their best. Mean reaction times for correct responses and error rates were subjected to two-way repeated measures analyses of variance (ANOVA) with factors mask type (face mask, grey mask) and priming (primed, unprimed). Trials for familiar and unfamiliar targets were analysed separately.

## Results

**Fig. 1:**
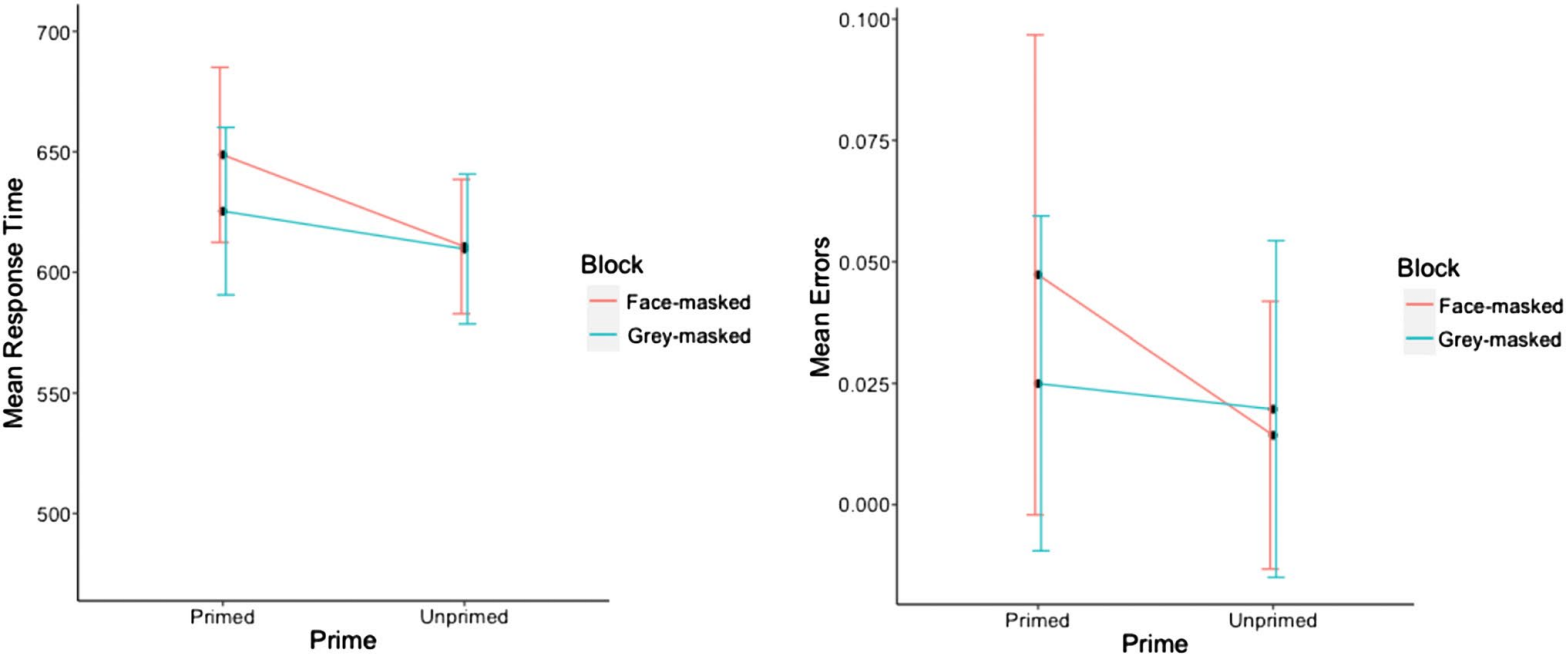
Mean response times and mean error rates for unfamiliar faces in Experiment 1

Reaction times to familiar faces (Fig. 1) showed a significant main effect of priming, *F*(1, 27) = 141.94, *p* < 0.001, ${\eta }_{p}^{2}$ = 0.84, with participants responding faster on primed trials (*M* = 563 ms, *SD* = 105) than on unprimed trials (*M* = 640 ms, *SD* = 104). The main effect of mask type was non-significant, *F*(1, 27) = 0.02, *p* = 0.89, ${\eta }_{p}^{2}$ < 0.01; there was, however, a significant interaction between factors priming and mask type, *F*(1, 27) = 14.25, *p* < 0.001, ${\eta }_{p}^{2}$ = 0.35. In assessing the extent to which the mask affected priming of familiar faces, follow-up *t*-tests revealed significant positive priming effects for both face-masked, *t*(27) = − 7.67, *p* < 0.001, and grey-masked trials, *t*(27) =  − 12.42, *p* =  < 0.001, with the magnitude of the priming effect being larger for face-masked trials (94 ms) than for grey-masked trials (61 ms).

The analysis of error rates for familiar faces showed a significant main effect of priming, *F*(1, 27) = 4.30, *p* = 0.048, ${\eta }_{p}^{2}$ = 0.14, with participants committing more errors on unprimed trials (*M* = 0.05, SD = 0.08) than for primed trials (*M* = 0.04, SD = 0.07). The main effect of the mask type was not significant, *F*(1, 27) = 0.89, *p* = 0.36,${\eta }_{p}^{2}$= 0.03, nor was the interaction between the two factors, *F*(1, 27) = 0.92, *p* = 0.35, ${\eta }_{p}^{2}$ < 0.01.

Reaction times for unfamiliar faces revealed no significant main effect of mask type, *F*(1, 27) = 1.28, *p* = 0.27, ${\eta }_{p}^{2}$ = 0.05. The main effect of priming was significant, *F*(1, 27) = 19.77, *p* < 0.001, ${\eta }_{p}^{2}$ = 0.42, with participants being slower to respond on primed trials (*M* = 638 ms, *SD* = 115) than for unprimed trials (*M* = 610 ms, *SD* = 101). The interaction between the priming and mask condition was also significant, *F*(1, 27) = 8.65, *p* = 0.007, ${\eta }_{p}^{2}$ = 0.24. Follow-up pairwise comparisons showed a significant reversed priming effect for face-masked trials only, *t*(27) = 5.65, *p* < *0.0*01. For grey-masked trials, this comparison was not significant, *t*(27) = 1.20, *p* = *0.1*1.

Error rates for unfamiliar faces revealed a non-significant effect of mask type on the percentage of incorrect responses, *F*(1, 27) = 2.95, *p* = 0.097, ${\eta }_{p}^{2}$= 0.13. The main effect of priming was significant, *F*(1, 27) = 9.42, *p* = 0.005, ${\eta }_{p}^{2}$ = 0.22, with participants committing more errors for primed trials (*M* = 0.04, *SD* = 0.04) than for unprimed trials (*M* = 0.02, *SD* = 0.03) indicating the presence of the reversed priming effect also in response accuracy. The interaction between priming and mask type was also significant, *F*(1, 27) = 4.21, *p* = 0.049, ${\eta }_{p}^{2}$ = 0.14. Follow-up analyses revealed a significant reverse priming effect in error rates for face-masked, *t*(27) = 3.46, *p* = 0.004, but not for grey-masked trials, *t*(27) = 0.60, *p* < 1.

## Discussion

The main aims of Experiment 1 were to replicate the reversed priming effect for unfamiliar faces and to determine the role of the face mask by comparing it with a grey ellipse. The results are informative in two ways. First, we successfully replicated the reversed priming effect with unfamiliar primed faces being responded to more slowly than unfamiliar unprimed faces when a face mask stimulus was presented between the presentation of the prime and target faces. Second, as predicted our results demonstrate the role of the intervening face mask in producing this effect: Presenting a grey mask as an intervening stimulus attenuated the reversed priming effect for reaction times. Please note, however, that replacing the face mask by an unstructured grey stimulus, did not yield a positive priming effect, ruling out that the reversed priming effect is due to masking alone.

An episodic memory retrieval account of priming can explain the effect of the intervening face mask in Experiment 1. The retrieval of the prime's memory trace during (unfamiliar) target processing allows participants to correctly attribute the increased target processing fluency to its previous presentation as the prime stimulus, rather than (incorrectly) to the earlier encoding phase. The intervening mask, which is similar to the prime—in that it is also a face stimulus—may disrupt the extent to which target processing fluency can be attributed to the prime in one of two ways. First, the mask may disrupt the consolidation or encoding of source information in the prime's memory trace, impeding the attribution of increased target processing fluency due to incomplete information in the trace for the prime. This idea is in line with the “mask-triggered inhibition” hypothesis (Jaśkowski, 2007; Jaśkowski & Przekoracka-Krawczyk, 2005), where an intervening mask stimulus inhibits prime processing. We refer to this explanation as the source consolidation account.

Alternatively, the mask stimulus may interfere with retrieval attempts that take place during target processing. Prime stimuli and face mask stimuli are perceptually similar and presented in rapid temporal succession; thus, the temporal proximity and similar features associated with both stimuli may lead to ambiguity during prime retrieval. If, as previously stated, participants retrieve the encoding episode of the prime stimulus upon target presentation and attribute increased processing fluency to that context, the mask stimulus may also be retrieved along with the prime trace due to its shared context. In this scenario, participants have to choose between two different episodic traces in order to attribute processing fluency, the prime and the mask stimulus, slowing reaction times to the target. However, when an irrelevant stimulus, sharing no features with the task-relevant (face) stimulus, such as the grey oval, is presented as an intervening mask, only the prime’s memory trace is retrieved upon target presentation, allowing for a quick matching with the target and faster attribution of the target's increased fluency to the prime. We refer to this explanation as the source retrieval account.

## Experiment 2

In Experiment 2 we aimed to (1) distinguish between the source consolidation and the source retrieval accounts of the reversed priming effect by enhancing the context segregation of the prime from the mask, (2) reduce the contribution of long-term memory processes to the priming task and (3) address the influence of memory consolidation in producing the reversed priming effect.

### Aim 1: contextual similarity of the mask

Previous studies have shown that in certain circumstances, a shared context between prime and target stimuli is essential for the prime's memory trace to be retrieved (Kahneman et al., 1992). To enhance the shared context of prime and target, we manipulated the contextual similarity of the face masks by presenting them inside a red frame, distinguishing the masks from both primes and targets. We reasoned that this should have two effects, first, the presentation contexts for the prime and mask stimuli would become less similar, and secondly, the contexts for the target and the prime would become more similar relative to that of the target and the mask. Crucially, the mask stimulus still consisted of a face. Therefore, the source consolidation and the source retrieval accounts of the reversed priming effect make different predictions. For the source consolidation account, the continued use of a face mask stimulus should disrupt the consolidation of the source information for the prime in a similar manner to an unframed face mask, resulting in a null effect of the frame on the reversed priming effect. On the other hand, the source retrieval hypothesis predicts that the contextual segregation between the prime and the mask should direct participants' retrieval efforts more easily to the prime stimulus, resulting in a faster comparison between the prime and the target. Therefore, target fluency should be attributed more quickly to the prime stimulus and, hence, the frame should significantly reduce the reversed priming effect.

### Aim 2: influence of long-term memory retrieval

We have suggested that the reversed priming effect is due to the interplay of conflicting signals based on long-term episodic memory retrieval and short term repetition priming. A second aim of experiment 2 was to test this idea by reducing the influence of long-term memory retrieval during the priming task. To investigate this we replaced the familiarity decision task with a matching task where participants were required to indicate if the target face was the same or different from the preceding prime face. We reasoned that this change in task would remove the contribution of long-term memory retrieval processes because participants could carry out this matching task based on short-term memory alone, and thus the reversed priming effect would be reduced.

### Aim 3: influence of memory consolidation

As previously discussed, it is likely that evaluating the familiarity of a famous face relies on semantic memory, whereas evaluating an experimentally learned face may be more reliant upon the episodic retrieval of the learning context and its associated perceptual features. We propose that whenever participants carry out familiarity judgments, the prime stimulus creates a sense of processing fluency for unfamiliar faces on a level that is largely irrelevant for successfully evaluating the familiarity of famous faces, which instead relies on the retrieval of semantic/biographical information. However, when evaluating a previously unfamiliar face as experimentally learned or new, the prime stimulus may increase processing fluency on the same level of processing as that required to carry out the familiarity decision, and therefore, participants may miss-attribute their experience of increased processing fluency to an earlier study period. To test this prediction, we compared two conditions where familiar targets were either 10 famous faces or a set of previously learned faces. We expected that the reliance on semantic memory for carrying out familiarity judgements on famous faces should attenuate the reversed priming effect for unfamiliar faces when evaluated in the same task context.

## Methods

### Participants

Twenty-one psychology students (10 female, age: *M* = 25.2 years, range: 21- 31, *SD* = 8) participated either for 8 € per hour or student credit points; 19 participants were right-handed as assessed by a handedness questionnaire (Oldfield, 1971). All participants reported normal or corrected-to-normal visual acuity and signed consent before the experiment. One participant did not complete the experiment.

### Design

Each of the three aims was manipulated in a block-wise design. Block A was identical to the first block in Experiment 1 aiming to again replicate the reversed priming effect as reported by Kaltwasser et al. (2014), and as replicated in Experiment 1. Block B examined our second aim of removing the influence of long-term memory processes during the priming task. Here we used the same materials as in Block A but changed the experimental instructions by asking participants to indicate if the target face was the same or different from the preceding prime face. Block C looked into our first aim which was to distinguish the context of the mask presentation from the target by displaying the intervening face mask stimulus within a red frame, using the same 10 familiar faces and 40 novel unfamiliar faces. Finally, Block D aimed to investigate our third aim; here we replaced the 10 familiar faces from the learning phase with a set of 10 famous faces which were intermixed with 40 novel unfamiliar faces. Block order was counterbalanced across participants. In Blocks A, C, and D, the task was to categorise the target stimuli as unfamiliar or familiar by button presses.

### Materials

The same set of materials as in Experiment 1 was employed, with the exception of the grey mask. Additionally, framed trials in the first block displayed a red oval frame placed around a new set of unfamiliar faces that served as face mask stimuli. Two raters selected the 10 best-known faces from the set of famous faces used by Dörr et al. (2011) for presentation in the fourth block.

### Procedure

The experimental procedures were the same as those in Experiment 1.

## Results

### Aim 1: comparing face masks with framed face masks

**Fig. 2:**
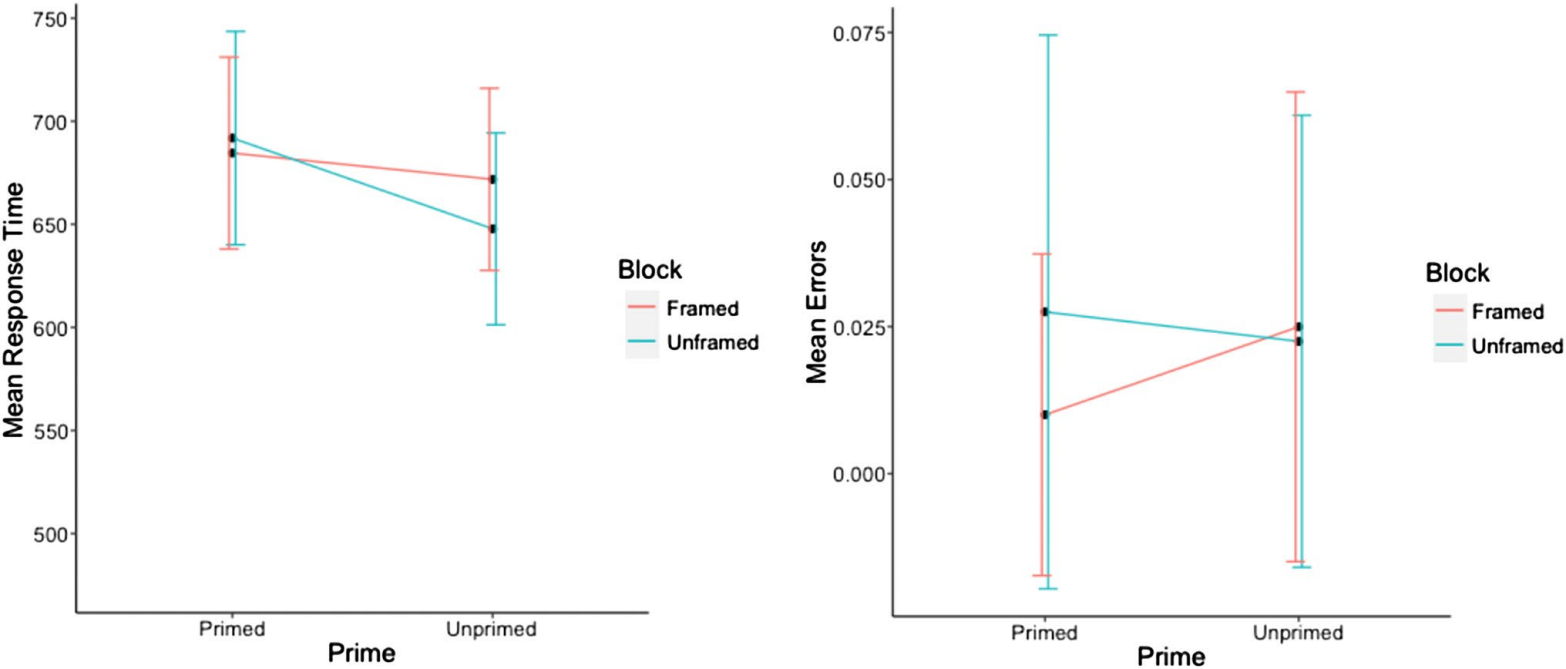
Mean response times and mean error rates for unfamiliar faces in Experiment 2 (aim 1)

Reaction times and error rates for familiar and unfamiliar targets were submitted to repeated measures ANOVAs with factors priming (primed, unprimed) and frame (framed, unframed). Mean results per condition are presented in Fig. 2.

The reaction times for familiar faces revealed a significant main effect of priming, *F*(1, 19) = 61.26, *p* < 0.001, ${\eta }_{p}^{2}$ = 0.76, with participants responding faster in primed trials (*M* = 593 ms, *SD* = 117) than in unprimed trials (*M* = 670 ms, *SD* = 111), illustrating typical priming effects for familiar faces. There was no main effect of frame, *F*(1, 19) = 1.65, *p* = 0.22, ${\eta }_{p}^{2}$ = 0.08, though the interaction between priming and frame was significant, *F*(1, 19) = 5.1, *p* = 0.036, ${\eta }_{p}^{2}$ = 0.21. Follow-up tests showed a significant effect of priming for both unframed trials *t*(19) = 6.35, *p* < 0.001, and framed trials *t*(19) = -7.35, *p* < 0.0001*,* with a larger priming effect in unframed than framed mask trials (92 ms vs. 62 ms).

The ANOVA for the error rates for familiar faces revealed a significant main effect of priming, *F*(1, 19) = 12.23, *p* = 0.002, ${\eta }_{p}^{2}$ = 0.39, with participants committing less errors on primed trials (*M* = 0.03, *SD* = 0.17) than on unprimed trials (*M* = 0.04, SD = 0.23), and no main effect of frame, *F*(1, 19) = 3.54, *p* = 0.075, ${\eta }_{p}^{2}$ = 0.16. The interaction between the factors priming and frame was not significant, *F* < 1.

Reaction times for unfamiliar faces revealed a significant main effect of priming, *F*(1, 19) = 20.27, *p* < 0.001, *η*_*p*_^*2*^ = 0.52, where, in contrast to the typical effect of priming observed for familiar faces, participants were slower to respond in primed trials (*M* = 689 ms, *SD* = 140) than in unprimed trials (*M* = 660 ms, *SD* = 138). The main effect of frame was not significant, *F(*1, 19) = 0.13, *p* = 0.72, *η*_*p*_^*2*^ = 0.01. However, the interaction between the factors frame and priming was significant, *F*(1, 19) = 6.86, *p* = 0.017, *η*_*p*_^*2*^ = 0.27. Follow-up *t* tests showed a significant reversed priming effect for unframed trials (44 ms), *t*(19) = 5.61, *p* < 0.001, whereas for framed trials, there was no significant priming effect (14 ms), *t*(19) = 1.46, *p* = 0.32.

Error rates for unfamiliar faces showed no main effects for frame, *F*(1, 19) = 0.81, *p* = 0.39, ${\eta }_{p}^{2}$ = 0.04, nor priming,* F*(1, 19) = 1.35, *p* = 0.259, ${\eta }_{p}^{2}$ = 0.07. There was, however, a marginally significant interaction between the two factors, *F*(1, 19) = 4.38, *p* < 0.049, ${\eta }_{p}^{2}$= 0.19. Follow-up analyses showed no significant effect of priming for unframed trials, *t*(19) = 0.81, *p* = 0.43, whereas for framed trials the reversed priming effect was a trend with participants committing more errors on unprimed compared to primed trials, *t*(19) = -2.26, *p* = 0.072.

### Aim 2: comparing familiarity task with the matching task

**Fig. 3:**
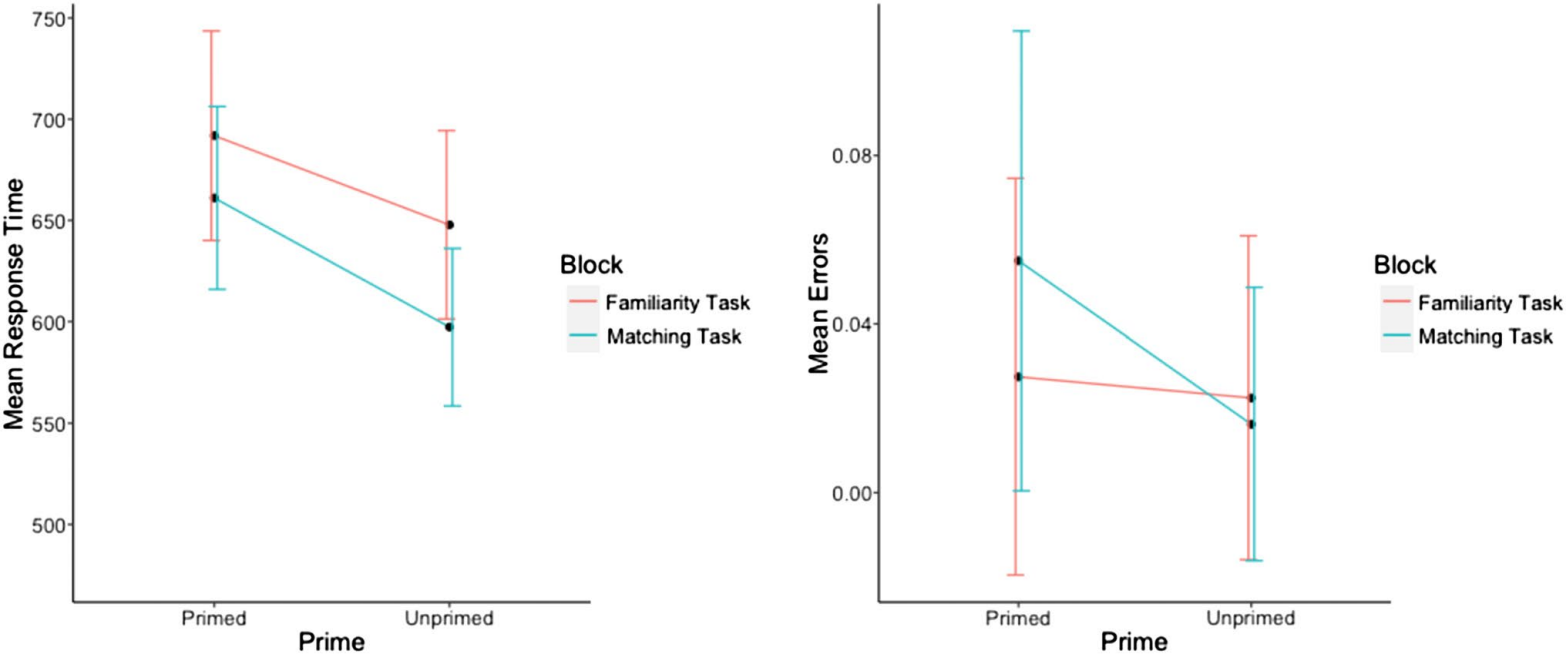
Mean response times and mean error rates for unfamiliar faces in Experiment 2 (aim 2)

Reaction times and error rates for familiar and unfamiliar targets were assessed with repeated measures ANOVAs with factors priming (primed, unprimed) and task type (familiarity task, matching task). Mean results per condition are presented in Fig. 3.

Reaction times to familiar faces (Fig. 3) showed a main effect of prime *F*(1, 19) = 82.8, *p* < 0.00001, ${\eta }_{p}^{2}$ = 0.81, a non-significant effect of task type *F*(1, 19) = 2.87, *p* = 0.11, ${\eta }_{p}^{2}$ = 0.13. and a non-significant interaction between the two *F*(1, 19) = 0.46, *p* = 0.51, ${\eta }_{p}^{2}$ = 0.02.

Error rates for familiar faces showed significant main effects of prime *F*(1, 19) = 12.9, *p* < 0.002, ${\eta }_{p}^{2}$ = 0.4 and task type *F*(1, 19) = 13.46, *p* < 0.002, ${\eta }_{p}^{2}$ = 0.42. The interaction between the two main factors was marginally non-significant *F*(1, 19) = 3.86, *p* = 0.06, ${\eta }_{p}^{2}$ = 0.17.

For unfamiliar faces the results showed a significant main effect of prime *F*(1, 19) = 36.6, *p* < 0.00001, ${\eta }_{p}^{2}$ = *0.6*7 and of task type *F*(1, 19) = 5.54, *p* < 0.03, ${\eta }_{p}^{2}$ = 0.23*,* the interaction between these two factors was non-significant *F*(1, 19) = 2.32, *p* = 0.14, ${\eta }_{p}^{2}$ = *0.1*1.

Mean error rates in responses to unfamiliar faces showed a significant main effect of prime *F*(1, 19) = 11.91, *p* < 0.003, ${\eta }_{p}^{2}$ = 0.39, and a non-significant main effect of task type *F*(1, 19) = 1.63, *p* = 0.08, ${\eta }_{p}^{2}$ = 0.39, and a significant interaction between the two *F*(1, 19) = 4.73, *p* < 0.05, ${\eta }_{p}^{2}$ = 0.2. Follow-up contrasts revealed a non-significant priming effect for the familiarity task *t*(19) = 0.65, *p* = 0.43, whereas for the matching task there was a significant reversal of the priming effect on error rates *t*(19) = 9.23, *p* < 0.007.

### Aim 3: comparing learned face targets with famous face targets

**Fig. 4:**
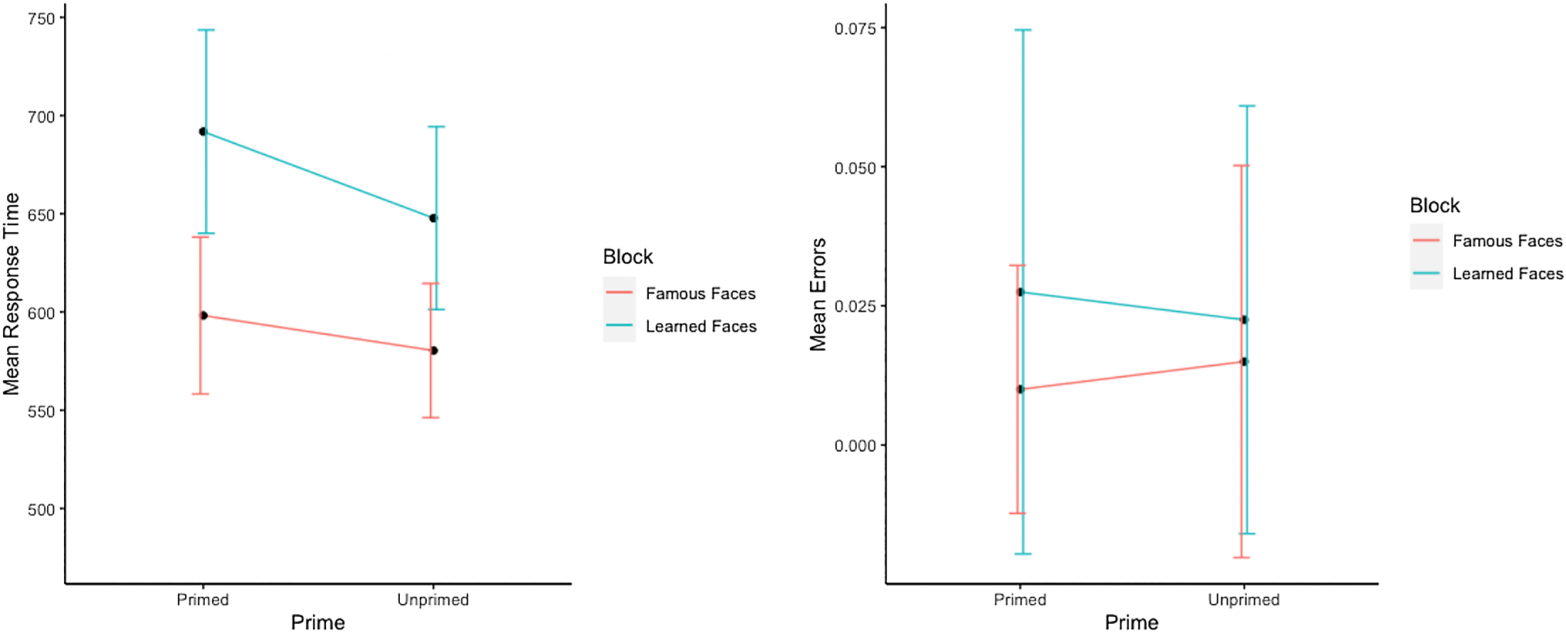
Mean response times and mean error rates for unfamiliar faces Experiment 2 (aim 3)

Reaction times and error rates for familiar and unfamiliar targets were assessed with repeated measures ANOVAs with factors priming (primed, unprimed) and consolidation (learned, famous). Mean results per condition are presented in Fig. 4.

For reaction times to familiar/famous faces the main effect of priming was significant, *F*(1, 19) = 66.46, *p* < 0.001, ${\eta }_{p}^{2}$ = 0.78, with participants being faster in primed than unprimed trials (*M* = 539 ms, *SD* = 108 vs. *M* = 629 ms, *SD* = 112), replicating standard positive priming effects. There was also a significant main effect of consolidation, *F*(1, 19) = 32.55, *p* < 0.001, ${\eta }_{p}^{2}$ = 0.63, with participants responding faster to famous faces (*M* = 545 ms, *SD* = 101) than to learned faces (*M* = 623 ms, *SD* = 123). The interaction between the two main factors consolidation and priming was not significant, *F* < 1.

In error rates for familiar/famous faces, there was no main effect of consolidation, *F* < 1. In contrast, the main effect of priming was significant, *F*(1, 19) = 6.57, *p* = 0.019, ${\eta }_{p}^{2}$ = 0.26, where, similarly to Experiment 2, participants committed less errors to primed faces (*M* = 0.02, *SD* = 0.16) than to unprimed faces (*M* = 0.03, *SD* = 0.20). The interaction between the factors priming and consolidation was not significant *F*(1, 19) = 1.64, *p* = *0.2*2, ${\eta }_{p}^{2}$= 0.08.

Reaction times for unfamiliar faces revealed a significant main effect of priming, *F*(1, 19) = 30.47, *p* < 0.001, ${\eta }_{p}^{2}$ = 0.62, where participants responded slower in primed trials (*M* = 646 ms, *SD* = 135) than in unprimed trials (*M* = 615 ms, *SD* = 130), a significant main effect of consolidation, *F*(1, 19) = 14.49, *p* = 0.001, ${\eta }_{p}^{2}$ = 0.43, with faster responses in the famous face block than in the learned face block (*M* = 590 ms, *SD* = 93 vs. *M* = 671 ms, *SD* = 153). There was also a significant interaction between the two factors, *F*(1, 19) = 9.91, *p* = 0.005, ${\eta }_{p}^{2}$ = 0.34. In exploring the nature of this interaction we found significant reversed priming effects for unfamiliar faces in both the learned block and the famous block, *t*(19) = 2.9, *p* = 0.018, with a larger reversed priming effect for unfamiliar faces in the learned block (44 ms vs. 17 ms).

The ANOVA of the error rates for unfamiliar faces revealed a significant main effect of consolidation, *F*(1, 19) = 5.59, *p* = 0.029, ${\eta }_{p}^{2}$ = 0.23, with participants committing more errors in response to learned faces v (*M* = 0.03, *SD* = 0.19) than to famous faces (*M* = 0.01, *SD* = 0.13) The main effect of priming was non-significant, *F* < *1*, nor was the interaction between the two factors, *F*(1, 19) = 1.85, *p* = 0.19,${\eta }_{p}^{2}$ = 0.09.

## Discussion

### Aim 1: contextual similarity of the mask

The results of Experiment 2 provide support for the source retrieval view. Presenting the face-mask stimulus in a red frame reduced the reversed priming effect in reaction times. We observed a reversed priming effect when the intervening face mask was presented in the same frame as the target and the prime stimuli, that is, when all three stimuli shared the same context. However, the difference between primed and unprimed conditions failed significance when the face mask was presented in a red frame, providing the mask with a distinct context relative to the prime and target stimuli. This result supports our hypothesis that the contextual similarity between the prime and the mask stimuli contributes to the creation of a conflict between memory signals, resulting in increased ambiguity regarding the source of target fluency for primed unfamiliar faces.

### Aim 2: influence of long-term memory

In comparing both reaction times and accuracy in the context of a matching task, participants still continued to show the reversed priming effect. These results run counter to our pre-experimental hypothesis. We reasoned that asking participants to focus on just the relation between the target face and its preceding prime, should reduce contributions from long-term memory retrieval and therefore reduce the reversed priming effect. However, the results showed the opposite pattern. Figure 3 shows that both the size of the reversed priming effect and the level of error rates were higher when responding to unfamiliar faces during the matching task. We believe that a closer examination of the error rates during the matching task offers an explanation for these results. Although we expected this condition to be the easiest one in the experiment, error rates for the conditions familiar-unprimed and unfamiliar-primed (11% and 6%) during the masking task were higher than in any of the other blocks in both Experiment 1 and Experiment 2. This suggests that the matching task was in fact the most difficult task. We reason that this difficulty is due to response interference in the matching task. Considering that in all other blocks, participants were required to make a familiarity decision, which requires a “yes” response for familiar faces, they are likely to have transferred the familiarity decision task to the matching block where for familiar-unprimed faces the correct response is “no”. Likewise, unfamiliar faces require a “no” response in the familiarity task but require a “yes” response in the primed condition in the matching task, resulting in response interference. We, therefore, suggest that despite our manipulation, participants were still experiencing interference in this block which makes it difficult to relate these results to broader aims of this paper.

### Aim 3: influence of memory consolidation

The comparison with famous faces as targets support our hypothesis regarding the role of memory consolidation in producing the observed reversed priming effects. We found that the use of famous faces as target stimuli in place of newly learned faces resulted in a significant reduction in the reversed priming effect for reaction times. These results illustrate the importance of a match between the levels of target processing, e.g., episodic information in case of newly learned faces and semantic information in case of famous faces. When the level of processing on which the prime stimulus exerts its effect is the same as the one where the participants carry out the familiarity judgment in order to evaluate the target stimulus, interference effects are strongest. However, when the effects of the prime operate at a different level than the one required to evaluate the target, interference effects are reduced.

## General discussion

In this study, we aimed to identify factors responsible for a reversed priming effect during the processing of unfamiliar faces in a repetition priming procedure first observed by Kaltwasser et al. (2014). We tested the hypothesis that the reversed priming effect is due to a conflict between different types of memory signals. In two experiments we replicated the reversed priming effect and found that three different features of the experimental design are responsible for eliciting the effect. First, the use of an intervening face mask between the prime and the target (face vs. grey ellipse); second, the contextual similarity of the mask (red frame vs. none); and third, the degree of memory consolidation of the familiar faces (famous vs. learned faces). Next, we will address each of these findings in turn.

### The effect of the mask

We have demonstrated that the use of the intervening masking stimulus is important in producing conflicts between memory representations. Experiment 1 showed that using a grey oval shape—instead of a new unfamiliar face—as a mask resulted in a reduction in the reversed priming effect for unfamiliar faces. This finding was further explored in Experiment 2 where we found that contextually segregating the prime and target stimuli from the mask by presenting the face mask within a red frame also reduced the reversed priming effect, which we suggest is due to the greater ease to attribute target processing fluency to the prime presentation. The contextual segregation of the mask may diminish the ambiguity regarding which previously presented face was the prime stimulus as participants are able to associate the prime stimulus to its context. This means that, when attempting to locate the source of increased target processing fluency, participants can focus their retrieval on the prime stimulus, allowing a faster comparison of the target stimulus with the prime, and thus participants can more easily attribute the increase in target fluency to the prime.

An interesting alternative explanation for the influence of presenting an intervening face mask on the reversed priming effect is that it disrupts the integration of the prime stimulus with the target. Evidence for the role of intervening events in disrupting the integration of two stimuli was found by Lupiáñez et al. (2013) who showed that presenting an intervening event between a cue and a target disrupted the integration of both stimuli and reinstated inhibition of return in a condition where it is not usually observed. An issue with this account for the current study, however, would be in explaining why placing the intervening masking stimulus in a red frame would differentially disrupt prime target integration.

A reviewer argued that since our study did not collect an independent measure of fluency, we cannot conclude whether the red frame manipulation facilitates the attribution of fluency to primes or disrupts fluency. An alternative explanation could be that perceptual fluency accruing from processing face primes was disrupted from the following target face by presenting a visual stimulus of high perceptual distinctiveness such as a red frame. This is an interesting point that could be a topic of futures studies.

### The effect of memory consolidation

The use of famous target faces resulted in a successful modulation of the reversed priming effect with reduced reaction times in comparison to experimentally learned faces. We argue that within the context of learned faces, participants rely on the retrieval of long-term episodic memory from the learning phase, whereas in the context of famous faces, familiarity judgments rely on accessing semantic information. It is this qualitative difference in the type of information used to solve the task that may in part be responsible for the occurrence of the reversed priming effect. When using learned faces, increased fluency may be mistaken for episodic familiarity resulting in a conflict with the results of the episodic retrieval process, whereas in a context of famous faces, increased processing fluency operates at a level that is irrelevant for the evaluation of the face where participants are instead relying on semantic memory. We would, however, like to point out that the relationship between processing fluency, episodic memory, and semantic memory is likely to be more complex with some studies showing that an increase in processing fluency may influence semantic judgements (Jacoby et al., 1989).

The reversed priming effect for famous faces was still present, albeit with a smaller magnitude (around half) than for learned faces. We tentatively suggest that this may be because participants develop a tendency to adopt an episodic retrieval strategy when evaluating famous and non-famous target faces. The famous target faces come from a set of 10 stimuli, which remained unchanged during the block. Thus, it may be possible that during the course of the experimental block, participants became familiar with the set of 10 famous faces and retrieved them when evaluating the target, comparing the target to the memory set, in a similar fashion to learned faces in the other blocks. The adoption of such a strategy would induce a conflict in the processing of unfamiliar primed faces, where increased processing fluency may cause participants to engage in the retrieval of episodic information. Despite this possibility, our significant reduction in reaction times supports a modulating role of target consolidation.

We have suggested an explanation for the reduction in the reversed priming effect when using famous faces in terms of the level of processing where the prime stimulus exerts its influence. An alternative explanation for the results of Experiment 2 can be given in terms of increased levels of consolidation for famous faces in comparison to learned faces. As stated in the introduction, people have extensive experience in recognising famous faces, and therefore, these faces should be highly consolidated in memory. Learned faces, on the other hand, should be substantially less consolidated in memory as they were presented for the first time on the same day of testing and thus participants' experience in recognising this set of stimuli should be minimal when compared with famous faces. Therefore, the effect of famous faces on the reversed priming effect may be due to their quantitative increase in memory strength.

### Memory conflicts

In two independent experiments, we replicated the reversed priming effect observed by Kaltwasser et al. (2014). This finding supports our hypothesis that the reversed priming effect emerged because signals from disparate memory processes—repetition priming induced fluency and episodic retrieval—may, under some circumstances, come into conflict. Participants' experience of increased fluency when processing unfamiliar primed target faces, caused participants to attribute target processing fluency to an earlier encoding phase; however, the outcome of episodic retrieval processes, indicated that the target face was not part of the learned memory set, and thus resulted in conflict between these opposing types of information arising from the target stimulus.

The results from this study are important for understanding models of human memory. One influential model of human memory conceptualises repetition priming and episodic memory as reflecting different types of memory. Specifically, repetition priming is thought to reflect a type of unconscious memory known as implicit memory, whereas episodic retrieval engagement during recognition testing reflects a type of conscious memory referred to as explicit memory (Graf & Schacter, 1985; Schott et al., 2005). It has further been postulated that implicit processes related to repetition priming and explicit processes related to episodic retrieval reflect separate memory systems (Schacter & Tulving, 1994; Tulving & Schacter, 1990). A conflict effect between repetition priming and episodic retrieval as found in the current study is theoretically important for memory models which propose that implicit and explicit forms of memory rely on separate processes. Completely independent processes require stochastic independence, involving “*complete absence of…overlap [of information, stages, processes, mechanisms]*” (Tulving, 1985, p. 295). The observation that information from these two processes may come into conflict suggests an interplay of the two types of memory and contradicts a strictly independent view. Our data speak more strongly in favour of interactive accounts of implicit and explicit memory processes (Cabeza & Moscovitch, 2013; Ferbinteanu, 2019). In keeping with an implicit/explicit approach to describing memory, an alternative explanation of the results from the current study is that the conflict might emerge between two sources of explicit memory, one triggered by the prime and the other by the target. Explicit memory of the prime, when repeated in the (unfamiliar) target might have occurred because the primes were not subliminal in our conditions. Hence, it is possible that a prime repeatedly shown as the subsequent target might have triggered not just fluency but indeed explicit recognition. If the target was unfamiliar and did not belong to the learned memory set, conflict would arise between explicit recognition and a signal that the target was not part of a memory set. Although this account is an option, we nevertheless lean towards an account of a conflict between explicit and implicit memory because the prime was not task relevant and was to be ignored. Future research with subliminal primes might clarify this question.

An alternative framework for describing the differences between repetition priming effects and episodic retrieval during recognition tasks is that they reflect a distinction between automatic and controlled processing (Hasher & Zacks, 1979). Repetition priming effects are types of automatic processing, whereas episodic memory retrieval would be an instance of controlled, or intentional processing. According to some theories (Jacoby et al., 1993, 1997), automatic and controlled processes reflect independent sets of processes. The current observations of conflict effects occurring between an automatic process such as repetition priming and a controlled process such as episodic retrieval—which as we have put forward are partly due to the misattribution of prime induced processing fluency to a previous study episode—speaks against the position that automatic and controlled processes are strictly independent (Yonelinas & Jacoby, 2012).

An further alternative explanation for the conflict effects that we observed in the current study is that rather than reflecting a conflict between different types of memory, they instead reflect a conflict at a later decision making stage where the repetition priming process results in an old response whilst the episodic retrieval process results in a new response for primed unfamiliar faces, and these two contradicting decision responses come into conflict. We cannot confidently conclude from the present results whether the conflict effects that we observed arise from a competition between different memory representations or between the decision outcomes of different memory retrieval processes; this question may be subject to future research.

## Conclusions

The present study replicated and further investigated the reversed priming effect reported a previous study (Kaltwasser et al., 2014). The results from several experimental manipulations support our explanation that the effect is related to the misattribution of prime induced processing fluency to the encoding episode rather than to the prime stimulus. It will be of interest to follow up this interplay under different conditions and across stimulus domains.

### Supplementary Information

Below is the link to the electronic supplementary material.

Supplementary file1 (DOCX 14 KB)

## Data Availability

Raw subject files and analysis scripts can be accessed at https://osf.io/z72rx/
