# Supplementary material for: Conflicts between priming and episodic retrieval: a question of fluency?
Source: Psychol Res. 2024 Feb 28;88(4):1127–40. doi: 10.1007/s00426-023-01919-4 (PMC11142949; doi:10.1007/s00426-023-01919-4)
Supplement: Supplementary file 1 — Supplementary file1 (DOCX 14 KB) [file 426_2023_1919_MOESM1_ESM.docx]

**Supplementary material**

In both experiments reported here we sought additional evidence for the presence of conflicts underlying the reversed priming effect by recording event-related potentials (ERPs). In the study of Kaltwasser et al. (2014) ERPs to primed relative to unprimed unfamiliar targets showed a sustained negativity, starting from 250 ms across frontal-central-parietal regions, similar in topography and timing to the N450 ERP component, a neurocognitive index of conflict detection commonly observed for incongruent trials in the Stroop task (Coderre, Conklin, & van Heuven, 2011; West & Alain, 1999). This finding stands in contrast to studies where familiarity decisions are required for famous versus unfamiliar faces. In such studies one usually finds a (small) posterior N250r and a later posterior negativity (LRE or N400) (e.g. Herzmann et al., 2004; Pfütze et al., 2002; Schweinberger et al., 1995). However, from hindsight, a late anterior negative priming effect around 400 ms can also be seen for unfamiliar faces in Figure 3 (bottom panel) of Herzmann et al. (2010) who had also required familiarity decisions between learned and unfamiliar faces and, similarly, in Herzmann and Sommer (2007) for faces and also names. The similarity of the timing and topography of the negativities reported in Herzmann & Sommer (2007), Herzmann et al. (2007) and Kaltwasser et al. (2014) and that elicited during Stroop tasks (e.g. Zahedi et al. 2019) indicates that the anterior negative effect reflects the processing of a conflict.

In the present studies, we expected to replicate these N450 effects; moreover we expected that ERPs would help to understand any modulations of the reversed priming effects due to our experimental variables. Specifically, we expected that the N450 would diminish to the extent that our manipulations would reduce or eliminate the reverse priming effect. Should behavioural modulation not be accompanied by ERP effects, it might indicate that the performance outcome is modulated in later stages, following those underlying the N450 effect.

**EEG Methods**

**EEG acquisition.** EEG was recorded (500 Hz sampling rate) from 40 Ag/AgCl electrodes mounted in an electrode-cap (Easycap GmbH) according to the extended 10–20 system using a left mastoid reference electrode, with AFz serving as ground. Impedance was kept below 5 kΩ, using ECI electrode gel (Expressive Constructs Inc., Worcester, MA). All channels were filtered online with a band pass of .05–70 Hz. Offline, the continuous EEG was down-sampled to 250 Hz. Blinks and eye movement artifacts were removed via independent component analysis as implemented in Brain Vision Analyzer software (Version 2.0. Brain Products, München, Germany). The continuous EEG was low-pass filtered at 30 Hz and 12 dB/octave roll-off, recalculated to average reference, segmented into -200 to 700 ms epochs relative to target onset, and referred to a 200-ms pre-stimulus baseline. Segments with amplitudes exceeding +/-200 μV, voltage steps > 100 μV per sampling point, and a voltage range > 300 μV were excluded. EEG epochs with correct responses only were averaged separately for each experimental condition.

Epoch boundaries for the early (ERE) and late (LRE) repetition effects for familiar faces were first determined as the point in time at which waveforms for primed and unprimed conditions began to differ at key electrode sites. This was then corroborated by visual inspection of the global map dissimilarity (GMD, Brandeis, Naylor, Halliday, Callaway, & Yano, 1992) for the difference of primed minus unprimed for familiar faces. GMD provides a measure of difference in scalp topographies between temporally adjacent maps, and thus may be used to demarcate periods of relatively stable topographies. ERE and LRE components were determined separately for each experimental block. Regions of interest for the ERE consisted of electrodes FC1, FC2, Cz, CP1 and CP2, and for the LRE electrodes C3, Cz, C4, CP1, CP2, P3, Pz and P4.

In analysing the influence of our experimental manipulations on ERPs for unfamiliar faces, we based time windows and regions of interest for the N450 component on those reported by Kaltwasser et al. (2014), which showed an early fronto-central negativity between 350 and 450 ms, centered around electrodes FC1, FC2, Fz, Cz, F3 and C3, and a later central parietal negativity between 450 and 650 ms, at electrodes CPz, Cz, CP1, and CP2. For each time window a two-way repeated measures ANOVA was conducted with factors mask type (face mask, grey mask) and priming (primed, unprimed). All follow-up comparisons are Bonferroni corrected and two-tailed unless stated otherwise.

**EEG Results Experiment 1**

**Familiar faces.** Examination of difference waves and GMD revealed differences at 288, 360 and 488 ms for face masked trials, and 268, 352 and 468 for grey masked trials. These values were then used as the boundaries for the ERE and LRE components.

***ERE.*** The analysis revealed significant main effects of mask type, *F*(1, 27) = 16.82, *p* < .001, $\eta_{p}^{2}$ = .38, and priming, *F*(1, 27) = 30.39, *p* < .001, $\eta_{p}^{2}$= .53, and a non-significant interaction between the two factors *F* < 1.

***LRE.*** The ANOVA for the LRE showed a significant main effect of priming *F*(1, 27) = 55.10, *p* < .0001, $\eta_{p}^{2}$ = .67. The effect of mask type and the two-way interaction were not significant, *Fs* < 1.

**Unfamiliar faces.** In the interval between 350 and 450 ms a frontal negativity can be seen for primed relative to unprimed faces, followed by a late positivity that was larger for primed than unprimed trials, especially at centro-parietal sites.

The amplitude of the early component showed a significant main effect of mask type, *F*(1, 27) = 4.53, *p* = .043, $\eta_{p}^{2}$= .14, a significant main effect of Prime, *F*(1, 27) = 10.24, *p* = .004, $\eta_{p}^{2}$ = .28, and a non-significant interaction between the two factors, *F*(1, 27) = 2.00, *p* = .17, $\eta_{p}^{2}$ = .069.

The analysis of the late component showed a significant main effect of Prime, *F*(1, 27) = 24.45, *p* <.001, $\eta_{p}^{2}$ = .48. No main effect of mask type was observed, *F*(1, 27) = .06, *p* = .80, $\eta_{p}^{2}$ = .002, but again the interaction between the factors mask and priming was not significant *F*(1, 27) = 1.86, *p* = .18, $\eta_{p}^{2}$ = .07.

**EEG Results Experiment 2**

**Analysis for Aim 1**

**Component** boundaries were identified by means of GMD at 216, 296, and 472 ms for unframed trials, and 224, 332, and 480 ms for framed trials. Comparison of ERE and LRE topographies revealed significant differences for both unframed, *F*(39, 741) = 9.72, *p* < .001$,\eta_{p}^{2}$ = .339, and framed trials *F*(39,741) = 7.835, *p* < .001, $\eta_{p}^{2}$ = .292.

**ERE.** The analysis revealed a significant main effect of frame *F*(1, 19) = 5.57, *p* < .03, $\eta_{p}^{2}$ = .23, with the amplitude of the ERE being larger for framed trials than for unframed trials, a significant main effect of priming *F*(1, 19) = 12.49, *p* < .003,$\eta_{p}^{2}$= .40, with an overall larger ERE for primed trials compared to unprimed trials, and a non-significant interaction between the two factors *F*(1, 19) = 2.20, *p* = .15, $\eta_{p}^{2}$ = .10.

**LRE.** Results showed no main effect of frame, *F*(1,19 = 1.02, *p* = .33*,* $\eta_{p}^{2}$ = .05. A significant main effect of Priming *F*(1, 19) = 34.71, *p* < .001,$\eta_{p}^{2}$ = .65, with a larger LRE for primed trials than for unprimed trials. The interaction between frame and priming was non-significant *F < 1*.

***Unfamiliar faces*** A two-way analysis of variance on mean voltages in the 350 – 450 ms time window (early component) revealed a non-significant main effect of frame, *F*(1,19) = .70, *p* = .41,$\eta_{p}^{2}$ = .04, a significant main effect of priming, *F*(1, 19) = 15.35, *p* < .001, $\eta_{p}^{2}$ = .45, and no interaction between priming and frame, *F* < 1.

Analysis of the later time window showed a trend for a main effect of frame, *F*(1, 19) = 3.75, *p* = .07, $\eta_{p}^{2}$ = .17; a significant main effect of priming *F*(1, 19) = 9.38, *p* < .01,$\eta_{p}^{2}$ = .33, but no interaction between the two factors, *F* < 1.

**Analysis for Aim 3**

***Unfamiliar faces.*** Time windows, regions of interest and factors used for the analysis were the same as in the previous analyses. In the early component window for unfamiliar faces we found a significant main effect of consolidation, *F*(1, 19) = 9.89, *p* = .006, $\eta_{p}^{2}$ = .34, with more positive amplitudes for unfamiliar faces in the famous face block, a significant main effect of priming, *F*(1, 19) = 16.68, *p* <.001,$\eta_{p}^{2}$ = .47, and a non-significant interaction between the two factors, F < 1.

Results for the late component showed a trend for a main effect of consolidation, *F*(1, 19) = 3.05, *p* = .096, $\eta_{p}^{2}$ = .14, a significant main effect of priming *F*(1, 19) = 13.42, *p* = .003. $\eta_{p}^{2}$ = .41, and a non-significant interaction between the two factors F < 1.

**Discussion**

In three conditions, one from Experiment 1 and 2 from Experiment 2, we replicated the N450 component to primed vs. unprimed unfamiliar faces. These findings are in contrast to previously reported priming effects for unfamiliar faces but replicate the results of Herzmann and Sommer (2007), Herzmann et al. (2010), and Kaltwasser et al. (2014), which went along with the paradoxical behavioural priming effect. The N450 has typically been observed in conflict tasks, such as the Stroop task where incompatible versus compatible trials also elicit a centrally distributed negativity (e.g. Zahedi et al. 2019) In the present studies, the N450 can be considered to reflect the conflict between the fluency signal from the prime and the retrieval of the episodic trace.

Interestingly, we did not find any significant modulations of the N450 amplitude as a function of the experimental manipulations. Naturally this might be due to a lack of sensitivity of the ERPs relative to the performance measures which did show effects of the manipulations. Alternatively, the effects of our manipulations could arise only after the initial conflict has occurred, which in this interpretation would be unmitigated by our manipulations. Hence, our N450 might reflect the conflict in the semantic system persisting across all experimental variations. In contrast the demonstrable variations of the behavioural conflict manifestations might be due to effects on stages subsequent to the N450-eliciting stage, such as conflict detection or conflict resolution.
